# Effects of Hormone Replacement Treatment with Estrogen and Progestins on the Vascular Renin–Angiotensin System of Ovariectomized Rats

**DOI:** 10.3390/ijms26104930

**Published:** 2025-05-21

**Authors:** Laís Almeida Menezes, Patrick Wander Endlich, Deiviany Santana Santos Lima, A. Augusto Peluso, Simone Alves de Almeida, Mariana Veronez Borgo, Robson Augusto Souza Santos, Glaucia Rodrigues de Abreu

**Affiliations:** 1 Department of Physiological Sciences, Health Sciences Center, Federal University of Espírito Santo, Vitória 29043-900, Brazil; lais_a-m@hotmail.com (L.A.M.); simoalves@yahoo.com.br (S.A.d.A.); borgomv@gmail.com (M.V.B.); glaucia.abreu@ufes.br (G.R.d.A.); 2 Mucuri School of Medicine, Post-Graduate Program in Health Sciences, Federal University of the Jequitinhonha and Mucuri Valleys, Teófilo Otoni 39803-371, Brazil; 3 Faculty of Health and Medical Sciences, University of Copenhagen, 2300 Copenhagen, Denmark; augustopeluso@gmail.com; 4 Department of Physiology and Biophysics, Federal University of Minas Gerais, Belo Horizonte 31270-901, Brazil; robsonsant@gmail.com

**Keywords:** hormone therapy, estrogen and progestins, cardiovascular regulation, RAS

## Abstract

The renin–angiotensin system (RAS) is the main endocrine and tissular component responsible for controlling cardiovascular homeostasis, which can be modulated by estrogen levels. This study investigated the effects of hormone treatments with estrogen and progestins on angiotensin-(1-7)-mediated [Ang-(1-7)] vasodilation in ovariectomized rats and the possible mechanisms involving the RAS. Female Wistar rats were divided into the following groups: sham (SHAM), ovariectomized (OVX), OVX and treated with 17β-estradiol (E2) (OE_2_), OVX and treated with E2 and drospirenone (OE_2_ + DRSP), and OVX and treated with medroxyprogesterone (MPA). Hormonal treatment was delivered via gavage for 28 days. Vascular responses to Ang-(1-7) were assessed in isolated aortic rings, and a Western blot of the thoracic aorta was used to determine the protein levels of angiotensin II (Ang II) type-1 receptor (AT_1_R), Ang II type-2 receptor (AT_2_R), Ang-(1-7) receptor (Mas), angiotensin-converting enzyme 2 (ACE2), and endothelial nitric oxide synthase (eNOS). The results showed impaired vascular reactivity caused by ovariectomy. Ang-(1-7) induced vasodilation in the OE_2_, OE_2_ + DRSP, and MPA-treated groups, while the administration of the AT_2_R antagonist (PD123319) or the selective Mas antagonist (A779) increased the extent of vasorelaxation induced by Ang-(1-7) in the OVX + MPA group. There were no differences in the aortic levels of AT_1_R or ACE2 between the groups, but the MPA group showed significantly increased levels of AT_2_R and eNOS. We concluded that ovariectomy induced vascular dysfunction linked to RAS regulation, and both estrogen (E2) and progestins differentially restored these parameters.

## 1. Introduction

The incidence of cardiovascular diseases (CVDs) is sex-specific, as shown in the current literature [1,2]. While women of fertile age present a lower incidence of CVD than men of the same age [3], after menopause or bilateral ovariectomy, this protective cardiovascular profile is lost, and both sexes present similar chances of developing CVDs [3,4]. The CVD protection observed in young women is partially attributable to 17β-estradiol (E2), since menopause results in substantially decreased levels of this hormone [5].

E2 treatment in ovariectomized rats showed a positive correlation with cardiovascular benefits, including decreased blood pressure and protection against oxidative stress, endothelial dysfunction, and vascular remodeling [6,7]. Moreover, the first prospective observational clinical study using hormone replacement treatment (HRT) with estrogen, the Nurses’ Health Study (NHS), showed that postmenopausal HRT seems to decrease the risk of major coronary events in women without previous heart disease. However, estrogen replacement combined with progestin may also increase stroke risk [8]. Data from two other studies also indicated that HRT resulted in not only fewer cardiovascular benefits but also adverse effects, including ischemic stroke and thromboembolic events [9,10]. Thus, the effects of HRT on the human cardiovascular system are still controversial, and HRT combined with progestins (synthetic compounds with progestational properties similar to natural progesterone) represents a major alternative for further clarification [9,10,11,12].

The side effects caused by the administration of progestins are associated with the activation of steroid receptors, such as glucocorticoid, estrogen, and androgen receptors. In postmenopausal women, medroxyprogesterone acetate (MPA), a progestin with a high androgenic profile [13], reduced the effect of estrogen on endothelium-dependent relaxation [14]. Different animal models also showed that MPA treatment attenuated E2-induced vasodilation mediated by sodium nitroprusside [15], constricted coronary arteries, diminished endothelium-mediated dilation [16], and led to long-duration constrictions of epicardial coronary arteries [17]. Additionally, the coadministration of E2 and MPA in hypertensive ovariectomized rats abrogated the beneficial effects of E2 on blood pressure, cardiac hypertrophy, vascular osteopontin expression, perivascular fibrosis, and the NO-dependent relaxation of isolated aortic rings [18]. Interestingly, such deleterious effects were not observed for the administration of drospirenone (DRSP, a 17-α-spironolactone-derived progestogen with anti-androgenic effects [18] and the only synthetic progestin with anti-aldosterone properties) or E2 alone [19]. In fact, our research group showed that co-treatment with E2 and DRSP reduced blood pressure and improved bradykinin-mediated vasodilation in the coronary arterial beds of ovariectomized hypertensive rats [20].

E2 levels also influence hormonal pathways of cardiovascular control, such as the renin–angiotensin system (RAS), being capable of downregulating the vasoconstrictor axis of angiotensin-converting enzyme/angiotensin II/angiotensin AT_1_-receptor (ACE/Ang II/AT_1_R) and upregulating the vasodilator/protective axes of ACE2/angiotensin-(1-7)/Mas-receptor (ACE2/Ang-(1-7)/Mas) and Ang II/angiotensin AT_2_-receptor (Ang II/AT_2_R) [21,22]. In spontaneously hypertensive rats (SHRs), hypoestrogenism increased Ang II/AT_1_R-induced vasoconstriction [22] and cardiac ACE2 activity [23] while decreasing ACE2 activity in the kidneys [24]. However, elevated E2 levels in both intact and ovariectomized rats promoted vasodilation via Ang-(1-7)/Mas [25].

Although the effects of E2 are widely described, there is still a lack of studies exploring the vascular effects of this hormone treatment when combined with DRSP or MPA on the renin–angiotensin system (RAS). Therefore, this study examined the effects of a combined treatment of DRSP with E2 or MPA on Ang-(1-7)-dependent vasodilation and the possible mechanisms involving other RAS components.

## 2. Results

### 2.1. E2 and DRSP Treatments Prevent Body Mass Gain in Ovariectomized Rats

The initial and final body masses, body mass gain, uterus dry weight, uterus weight/tibia length ratio, and adipose depots were analyzed, and the data are summarized in Table 1. All animals presented a similar body mass at the beginning of the study. After 4 weeks, there was a significant body weight gain in OVX. This effect was prevented in OE_2_ and OE_2_ + DRSP but not in MPA, which presented greater adipose depots, indicating an increased total fat mass compared to the other groups.

The efficacy of ovariectomy was evidenced by a reduction in both the absolute uterus dry weight and the uterus weight/tibia length ratio in the OVX rats, which was prevented by all the hormonal treatments, despite not reaching the SHAM values. Lastly, both OE_2_ and OE_2_ + DRSP presented a greater uterus dry weight than OVX + MPA.

### 2.2. Hormonal Treatments Preserve Ang-(1-7)-Induced Vasodilation in an NO-Dependent Manner in the Aortic Rings of Ovariectomized Rats

**Figure 1:**
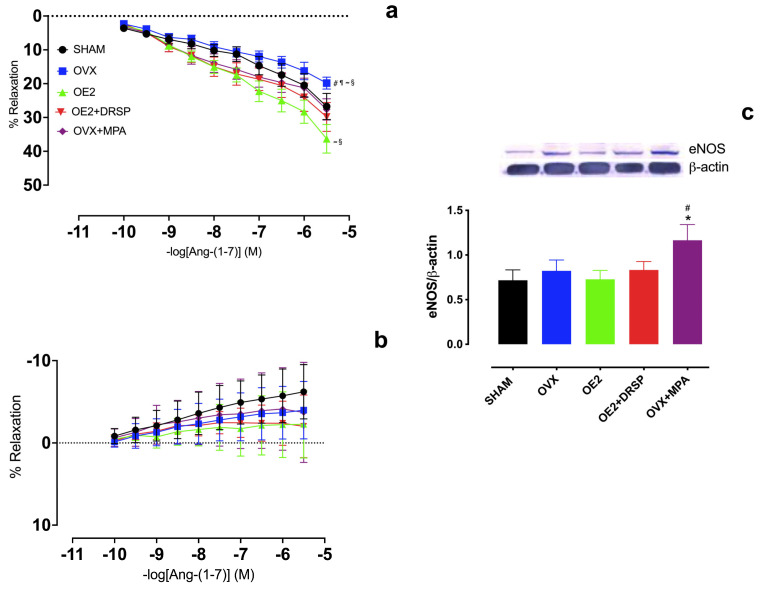
Influence of NOS inhibition on Ang-(1-7) vasodilation and eNOS levels in aortic tissue from ovariectomized Wistar rats subjected to different hormone therapies. (**a**) Dose–response curves to Ang-(1-7) in aortic rings (10^−10^–10^−5.5^ M) and (**b**) in the presence of L-Nitro^G^-L-Arginine Methyl Ester (L-NAME). Groups: SHAM (N = 11); ovariectomized (OVX, N = 10); ovariectomized with 17β-estradiol treatment (OE_2_, N = 14); ovariectomized with 17β-estradiol plus drospirenone treatment (OE_2_ + DRSP, N = 13); ovariectomized with acetate of medroxyprogesterone treatment (OVX + MPA, N = 13). (**c**) Representative Western blot figures and quantification of the treatment conditions for total eNOS levels normalized to ß-actin levels, used as loading control. Groups: SHAM, N = 5; OVX, N = 5; OE_2_, N = 5; OE_2_ + DRSP, N = 5; OVX + MPA, N = 5. Values are expressed as mean ± SEM. * *p* < 0.05 vs. OVX; ^#^ *p* < 0.05 vs. SHAM; ^¶^ *p* < 0.05 vs. OE_2_; ^≈^ *p* < 0.05 vs. OE_2_ + DRSP; ^§^ *p* < 0.05 vs. OVX + MPA. (**a**,**b**) Two-way or (**c**) one-way ANOVA, followed by (**a**,**b**) Tukey’s or (**c**) LSD post hoc test.

Intact endothelium-containing isolated aortic rings were used to evaluate the effects of estrogen deficiency and hormone treatments on the vascular responses to Ang-(1-7). As shown in Figure 1, ovariectomy (OVX) resulted in a significantly reduced dose-dependent vasodilation response to Ang-(1-7) compared to the other hormone treatments, indicating that estrogen deficiency impairs this mechanism. Furthermore, the lack of estrogen effect was prevented by either the combined OE_2_ + DRSP or MPA monotherapy (OVX + MPA). Interestingly, treatment with E2 alone (OE_2_) showed a greater improvement in Ang-(1-7)-mediated vasodilation responses (Figure 1a).

To determine whether the Ang-(1-7) vasodilator responses were mediated by nitric oxide (NO), Ang-(1-7) dose–response curves were assessed in the presence of L-NAME, a non-specific inhibitor of nitric oxide synthase (NOS). Ang-(1-7)-induced vasodilation was completely abolished in the presence of L-NAME in all groups, regardless of the type of hormone treatment (Figure 1b), indicating that NO plays a major role in the vascular responses induced by Ang-(1-7).

The effects of ovariectomy and hormone treatments on endothelial NO-synthase (eNOS) levels were assessed by Western blot. The total eNOS levels did not differ between the groups, except for OVX + MPA, which showed a significant increase in eNOS compared to SHAM and OVX (Figure 1c).

### 2.3. MPA, but Not DRSP Treatment, Preserves Ang-(1-7)-Induced Vasodilation Independently of AT_2_R and Mas Receptors in the Aortic Rings of Ovariectomized Rats

**Figure 2:**
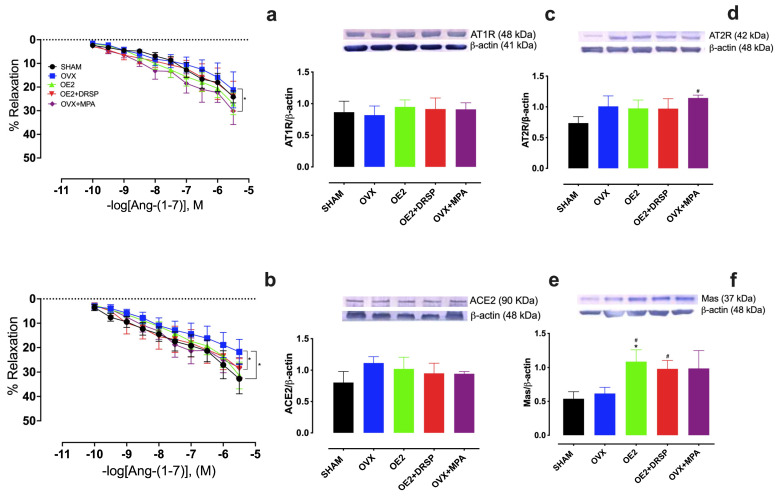
Effect of AT_2_R and Mas blockade on Ang-(1-7) vasodilation and RAS component levels in aortas of ovariectomized Wistar rats subjected to different hormonal treatments. Dose–response curves to Ang-(1-7) in aortic rings (10*^−^*^10^–10*^−^*^5.5^ M) in the presence of (**a**) PD123319 and (**b**) A779. Groups: SHAM (N = 8); ovariectomized (OVX, N = 9); ovariectomized with 17β-estradiol treatment (OE_2_, N = 9); ovariectomized with 17β-estradiol plus drospirenone treatment (OE_2_ + DRSP, N = 9); ovariectomized with acetate of medroxyprogesterone treatment (OVX + MPA, N = 8). Representative Western blot figures and quantification of the treatment conditions for (**c**) AT_1_R, (**d**) AT_2_R, (**e**) ACE2, (**f**) Mas levels to β-actin levels, used as loading control. Groups: SHAM, N = 5; OVX, N = 5; OE_2_, N = 5; OE_2_ + DRSP, N = 5; OVX + MPA, N = 5. Values are expressed as mean ± SEM. * *p* < 0.05 vs. OVX; ^#^ *p* < 0.05 vs. SHAM. (**a**,**b**) Two-way or (**c**,**d**,**e**,**f**) one-way ANOVA, followed by (**a**,**b**) Tukey’s or (**c**,**d**,**e**,**f**) LSD’s post hoc test.

We also evaluated the role of the AT_2_R [26,27,28] and Mas [29] receptors in mediating the vasodilation induced by Ang-(1-7) administration. For this, intact endothelium-containing isolated aortic rings were pre-incubated in the presence of the receptor antagonists PD123319 and A779, respectively (Figure 2a,b). Interestingly, Ang-(1-7)-induced vasorelaxation increased in OVX + MPA compared to in OVX in the presence of either PD123319 or A779. As expected, the ovariectomy (OVX) effect was demonstrated by the reduced vasodilatory responses to Ang-(1-7) compared to SHAM.

The levels of aortic AT_1_R, AT_2_R, Mas, and ACE2 in response to Ang-(1-7) were determined by Western blot. There were no differences in the AT_1_R (Figure 2c) or ACE2 (Figure 2e) levels between the groups. However, MPA showed increased AT_2_R compared to SHAM (Figure 2d). In addition, while both OE_2_ and OE_2_ + DRSP exhibited a significant increase in Mas levels compared to SHAM (Figure 2f), OE_2_ alone also showed higher levels of Mas than OVX (Figure 2f).

## 3. Discussion

This study evaluated the effect of a 4-week hormone treatment after ovariectomy in Wistar rats. Our main findings are summarized as follows: (i) E2, DRSP, and MPA treatments prevented reduced Ang-(1-7)-mediated vasodilation in the aorta of OVX rats; (ii) Ang-(1-7)-mediated vasodilation was increased in the aorta of OVX rats treated with MPA in a suggested AT_2_R- and Mas-independent manner; (iii) protective hormone therapies might have relevant involvement in NO-release, as well as in the regulation of RAS components.

The OVX and MPA-treated groups showed an increased body mass compared to all other groups, corroborating the literature data [30,31], suggesting that MPA administration, as commonly observed for progesterone, is capable of inducing a body mass increase by gestagenic actions [32]. A likely explanation for the body weight increase in ovariectomized animals can be related to an increase in hepatic or muscle fat, as previously shown [33]. However, such measurements were not captured in our study. In contrast, hormonal treatments with E2 and DRSP prevented the body mass gain caused by E2 deficiency [20,34,35]. Interestingly, clinical studies have shown that increases in body mass, the body mass index (BMI), fat deposition, and metabolic disorders caused by estrogen reduction and MPA treatment [36] were restored by replacement with E2 and drospirenone treatment [37,38,39]. The mechanisms for this include, for example, anti-mineralocorticoid activity and, consequently, decreased sodium reabsorption, followed by increased water excretion [40].

The RAS is an endocrine and local regulator of cardiovascular homeostasis and renal function [41]. Ang-(1-7) is a heptapeptide formed from alternative pathways to the classical ACE/AngII/AT_1_R axis, which presents vasodilatory properties, counteracting the vasoconstrictor actions of Ang II [41]. In fact, a reduction in estrogen levels in postmenopausal women or ovariectomy experimental models has been associated with hemodynamic and vascular dysfunctions due to RAS activation [21,22,42,43,44,45]. There is evidence that progestins, which are widely employed in HRT, may modulate the levels of Ang-(1-7) [46]. Consequently, understanding whether these hormones influence the vasodilatory responses mediated by Ang-(1-7) is essential for elucidating how they modulate blood pressure regulation and vascular function. This understanding is particularly relevant in contexts of hormonal fluctuations, such as during the menstrual cycle, pregnancy, or hormone therapy. This study showed an impaired Ang-(1-7)-mediated vasodilation response in the aorta of ovariectomized rats, similarly to our previous study conducted with hypertensive rats [45]. Therefore, hormonal treatments were able to attenuate decreased Ang-(1-7)-induced vasodilation, although only E2 treatment increased vasodilation to higher levels than SHAM.

Despite the observed beneficial effects described for DRSP and MPA treatments, there is no consensus in the literature. A study using rats ovariectomized by triptorelin showed that combined MPA + E2 treatment did not antagonize the beneficial effects of E2 [47]. In addition, MPA treatment alone did not induce significant changes in the arterial reactivity of the rats, measured by the pressure–diameter of the saphenous artery [47]. However, in our experimental setup, the ovariectomized rats receiving DRSP and MPA treatments were protected from the damage caused by estrogen deficiency, including the decreased vasodilatory response to Ang-(1-7).

The delivery of E2 via pellet implantation for 21 days showed an increased vasodilatory response to Ang-(1-7) in isolated mesenteric arteries of ovariectomized rats [25]. Furthermore, in OVX transgenic hypertensive rats, E2 treatment increased circulating levels of Ang-(1-7) and its vasodilatory responses, while the plasma levels of Ang II decreased [48]. In our study, treatment with E2 monotherapy improved Ang-(1-7)-mediated vasodilation in the aorta of the OVX rats, indicating that such a response is modulated by circulating levels of estrogen.

Ang-(1-7) vasodilation acts via its receptor Mas, but it has been shown to also interact with AT_2_R in mice and rats using both pharmacological and genetic approaches [49,50,51]. To evaluate these previous findings in our proposed model, dose–response curves were constructed in the presence of AT_2_R and Mas receptor antagonists, namely, PD123319 and A779, respectively. We expected that the use of both AT_2_R and Mas blockers would significantly reduce the vasodilatory response of Ang-(1-7). However, regardless of the group, incubation of the vessels with PD123319 or A779 resulted in a continued vasodilatory response to Ang-(1-7). Only the OVX + MPA group showed increased vasodilation compared to the OVX group, considering only the ovariectomized groups. In one of our previous studies, exogenous E2 did not alter Ang-(1-7)-induced vasodilatation in the aortic rings of ovariectomized SHRs blocked with A779. Nonetheless, when vessels were pre-incubated with PD123319, the vasodilatory effects of Ang-(1-7) were reduced [45]. We suggest that the divergence of data could be related to the route of administration (oral vs. subcutaneous) and mainly the dose of E2 treatment, time, or animal model, reinforcing the notion that the effects of E2 on enhancing the vasodilatory responses of Ang-(1-7) might be dose-dependent.

Oligomerizations/interactions between Ang-(1-7) and the receptors for Ang II (AT_1_R and AT_2_R) can trigger their functional actions [52,53]. In fact, it was found that the blockage of Mas with A779, PD123319, or CV11974 (an AT_1_R antagonist) did not change the vasodilatation induced by Ang-(1-7) in the aortic rings of male Sprague Dawley (SD) rats [54], suggesting the existence of a receptor subtype for Ang-(1-7) or that this response may depend on the vascular bed and/or animal species composition. Our data indicate that Ang-(1-7) may have exerted its responses via another G-protein-coupled receptor, such as the MrgD receptor, which is activated by Alamandine, an Ang-(1-7) breakdown peptide with vasodilatory properties [55]. Moreover, the inhibition of Ang-(1-7)-mediated vasodilation by PD123319 was observed only in the aortic rings of the rats treated with E2, suggesting that the selectivity of Ang-(1-7) by AT_2_R was E2-dependent [45,56].

Previous studies have shown that estrogen modulates the RAS via AT_2_R, causing a reduction in blood pressure associated with hypertension [30,57] and acting positively on congestive heart failure [58]. Contrary to our findings, a previous study by our research group showed that exogenous E2 did not alter the Ang-(1-7)-induced vasodilatation in the aortic rings of ovariectomized SHRs blocked with PD123319. We suggest again that the divergence of data could be due to the route of administration, dose, time of E2 treatment (28 days vs. 8 weeks), or animal model (Wistar rats vs. SHRs) [45].

To verify the mechanisms of action under the conditions of estrogen deficiency and the influence of hormonal treatment, we also investigated the protein levels of different RAS components and observed that the aorta of either the OVX rats or those treated with progestins and E2 for 4 weeks showed no differences in the levels of AT_1_R and ACE2 [21]. Contrarywise, previous studies demonstrated increased AT_1_R levels and AT_1_R mRNA overexpression in the aorta of OVX SD rats [31], which was reversed by estrogen treatment [59].

Increased ACE2 levels in the cardiac cells of an SD rat model of DOCA-salt of hypertension, as well as a preventive reduction in ACE2 in the kidneys of ovariectomized hypertensive SD rats, were found after treatment with E2 [60,61]. Our study was unable to show similar corroborative effects, which could simply be due to differences in the methodological approach, including antibody sampling and sensitivity; animal model; and time, dose, and pathways of the treatments studied. No additional data evaluating the effects of DRSP and MPA treatment on RAS components were found in the literature.

We showed greater levels of Mas in the aorta of ovariectomized rats treated with E2 alone or in combination with DRSP, which, in part, corroborates our functional results, since Ang-(1-7)-mediated vasodilation was restored in the hormone-treated OVX rats. In addition, no differences were observed in the aortic rings incubated with the Mas antagonist A779. This has been previously suggested to occur due to a possible receptor subtype for Ang-(1-7) or via Alamandine/MrgD, as mentioned above [62,63]. However, this was not part of the scope of this study and deserves further investigation. Lastly, no reference data were found for the results evaluating hormonal treatment with DRSP.

The vasodilatory effect of Ang-(1-7) is well known to be mediated by NO release [54,64] via the phosphorylation of endothelial nitric oxide synthase (eNOS) at different activation sites, such as at Ser1177 [65], or by increasing the total levels of eNOS. Upon Ang-(1-7) stimulation, eNOS is phospho-activated, increasing the synthesis of NO, which counter-regulates the vasoconstrictor Ang II/AT_1_R axis [66]. Our results showed that, in the presence of the NOS inhibitor L-NAME, the vasodilation responses were completely abolished in all experimental groups. Furthermore, we also observed increased eNOS levels in the aorta of the MPA-treated rats. Few studies have associated the RAS with MPA, and we could not find any study involving the regulation levels of AT_2_R or Mas. Our data suggest that the observed increase in AT_2_R could act as a compensatory mechanism for the possible functional endothelial impairment developed under MPA treatment; however, this was not evaluated in our study. Likewise, we observed an increase in eNOS levels, diverging from previous studies that demonstrated a reduction in the expression of this enzyme in endothelial cells [67,68]. This reinforces the hypothesis of a possible compensatory mechanism, as mentioned above.

A previous study showed that oxidative stress is increased in rats treated with MPA [69], which may lead to the uncoupling of eNOS, increasing its levels. However, it is important to point out that an increase in eNOS expression is not always accompanied by its overactivation, raising the need for additional studies to clarify such mechanisms.

### Clinical Perspectives

The clinical implications of this study are relevant due to the cardiovascular changes observed in hormonal treatments with E2 and MPA, which were capable of restoring Ang-(1-7)-mediated vasodilation. Early HRT in recently menopausal women was not linked to an increased risk of cardiovascular events but improved carotid intima–media thickness, an early indicator of atherosclerosis [70]. Our results on increased endothelial function via E2+DRSP suggest that correctly initiated HRT can lead to vascular benefits through safer and more effective treatments. Moreover, since conventional HRT has been associated with thrombotic and cardiovascular events in previous clinical investigations [71], our results suggest that choosing the right progestin type could significantly impact cardiovascular health.

## 4. Materials and Methods

### 4.1. Experimental Animals

Female Wistar rats weighing about 230 g and 10 weeks of age were provided by the Health Sciences Center facility of the Federal University of Espírito Santo. The animals were kept in collective cages with a regulated light cycle (12 h light/12 h dark), controlled temperature (22 °C to 24 °C) and humidity (40 to 60%), and free access to water and food (Purina Labina^®^, São Paulo, SP, Brazil). All procedures were conducted following the guidelines of the Guide for the Care and Use of Laboratory Animals and were approved by the Ethics Committee for Animal Use (CEUA) of the Federal University of Espírito Santos (UFES) (protocol 056/2016). At the time of ovariectomy, the animals were randomly divided into five groups: sham (SHAM), ovariectomized (OVX), OVX and treated with E2 (OE_2_), OVX and treated with E2 and drospirenone (OE_2_ + DRSP), and OVX and treated with medroxyprogesterone (MPA).

### 4.2. Ovariectomy

In the first 48 h following the acquisition of the animals, they were anesthetized with ketamine (80 mg/kg) and xylazine (12 mg/kg) and submitted to ovariectomy. A bilateral dorsolateral incision was made in the skin, and the underlying muscle was dissected to locate the ovaries and uterine tubes, which were ligated with a suture line for ovary removal [72]. Surgery but not ovariectomy was performed in the SHAM-operated rats [72]. At the end of the procedure, the rats received 0.1 mL of Enrofloxacin 2.5% intramuscular (IM) and Dipyrone Monohydrate for analgesia (100 mg/kg).

### 4.3. Hormonal Treatments

The hormonal treatment started after 14 days of post-operative recovery. The animals were orally treated by gavage once a day for 28 days. The hormones were dissolved in 0.1 mL of carboxymethylcellulose (CMC) and used immediately. The OE_2_ group received 0.075 mg/kg per day of E2 (Sigma-Aldrich, St. Louis, MO, USA) [73]. The DRSP group received 0.075 mg/kg per day of E2 (Sigma-Aldrich, St. Louis, MO, USA) + 0.15 mg/kg per day of drospirenone (Bayer Weimar, Weimar, Germany) [17], and the OVX + MPA group received 1 mg/kg per day of medroxyprogesterone acetate (SEM, São Paulo, Brazil) [74]. The SHAM and OVX groups received the same volume of vehicles [19]. The efficacy of the ovariectomy and experimental treatments was assessed by the weight of the dry uterus and the ratio between the uterine weight and tibia length [75].

### 4.4. Estrous Cycle

The estrous cycle was evaluated by vaginal smears in two consecutive cycles in the SHAM rats before sacrifice. The vaginal epithelial cells were collected daily between 08:00 h and 09:00 h and examined by light microscopy [76] for the identification of cell types in different phases of the estrous cycle. The rats were considered suitable for the experimental protocols during the proestrus stage, which is characterized by high estrogen levels, to avoid any influences of hormonal variation in different phases of the cycle [77].

### 4.5. Dissection and Vascular Reactivity of Isolated Thoracic Aorta Rings

After anesthesia with ketamine (80 mg/kg) and xylazine (12 mg/kg), all rats were sacrificed by decapitation. The chest was opened for the thoracic aorta removal and dissection in Krebs–Henseleit solution (KHS, in mM: 118 NaCl, 4.7 KCl, 2.5 CaCl_2_·2H_2_O, 1.2 MgSO_4_·7H_2_O, 1.2 KH_2_ PO_4_, 23 NaHCO_3_, Glucose 11, and 0.01 EDTA).

At the end of the study, the aorta was cut into cylindrical segments (rings) and subjected to reactivity experiments. Small rings were snap-frozen in liquid nitrogen and stored at −80 °C for further analysis. After isolation from connective and fat tissue, the samples were divided into six isolated rings of 3.5 to 4 mm in length. The aortic rings were mounted between two parallel wires in organ baths containing Krebs–Henseleit solution at 37 °C, pH 7.4, and they were then gassed with 95% O_2_ and 5% CO_2_. The aortic rings were stretched to an optimal resting tension of 1 g. Isometric tension was recorded using a force transducer (TSD125C, CA, USA) connected to an acquisition system (PowerLab^TM^, ADInstruments, Castle Hill, Australia). Following a 45 min equilibration period, all aortic rings were exposed twice to 75 mM KCl. Endothelial integrity was tested with acetylcholine (Ach, 10 μM) in the aortic rings previously contracted with phenylephrine (1 μM). A relaxation equal to or greater than 70% was considered demonstrative of endothelial functional integrity. After a 30 min washout period, the aortic rings were pre-contracted with phenylephrine (1 μM), and cumulative concentration–response curves with Ang-(1-7) (10^−10^ M to 2 × 10^−5.5^ M; Sigma, St. Louis, MO, USA) were constructed. Ang-(1-7) vasodilation properties were evaluated in the absence and presence of the following compounds: PD123319 (0.1 μM, AT_2_R antagonist; Sigma, St. Louis, MO-USA), A-779 (10 μM, selective Mas antagonist; Sigma, St. Louis, MO, USA), and L-Nitro^G^-L-Arginine Methyl Ester (L-NAME, non-specific nitric oxide synthase inhibitor, 100 μM, Sigma, St. Louis, MO-USA). The rings were incubated with inhibitors for 30 min before the curves were constructed, and responses were recorded using a data acquisition system (LabChart 8, ADInstruments, Pty Ltd., Bella Vista, NSW, Australia) coupled to a computer.

### 4.6. Western Blot

Samples were homogenized in a lysis buffer (in mmol/L: 150 NaCl, 50 Tris-HCl, 5 EDTA 2 Na, 1 MgCl_2_) and in a protease inhibitor cocktail from Sigma, St. Louis, MO, USA. The tissue homogenate was centrifuged at 4 °C and 14,000 RPM, the pellet was discarded, and the protein concentration was assayed by the Bradford method [78], using bovine serum albumin (BSA) as a standard. A total of 50 μg protein was electrophoresed (2:30 h 80 V) in 10% polyacrylamide gel (SDS-PAGE) and transferred to a polyvinylidene fluoride membrane (PVDF, Millipore, Darmstadt, Germany) for 1 h 40–2 h 30 at 60 V in a semi-dry blotting system. After blocking the membranes with TBS-T + 5% skim milk for 2:30 h, the samples were incubated overnight at 4 °C with the following primary antibodies: rabbit monoclonal anti-AT_1_R (1: 500, Santa Cruz Biotechnology, Waltham, MA, USA), rabbit polyclonal anti-AT_2_R (1: 500, Santa Cruz Biotechnology, Waltham, MA, USA), rabbit monoclonal anti-eNOS (1: 2500, BD Transduction Laboratories, Lexington, KY, USA), mouse monoclonal anti-ACE2 (1: 200, Santa Cruz Biotechnology, Waltham, MA, USA), and rabbit polyclonal anti-Mas (1: 500, Santa Cruz Biotechnology, Waltham, MA, USA). After washing with TBS-T, the membranes were incubated with the secondary antibodies according to the primary host: anti-mouse IgG (1: 2500, Sigma, St. Louis, MO-USA) or an anti-rabbit antibody (1: 7000, Sigma, St. Louis, MO, USA). Protein bands were visualized using an NBT/BCIP Kit (5-bromo-4-chloro-3-indolyl phosphate nitroblue tetrazolium (NBT)/5-bromo-4-chloro-3-indolyl phosphate (BCIP)—Invitrogen, Carlsbad, CA, USA) and quantified using ImageJ software, https://imagej.net/ij/ accessed on 17 September 2024 (National Institutes of Health, Bethesda, MD, USA). Protein levels were normalized by β-actin (monoclonal anti-mouse 1: 5000, Santa Cruz Biotechnology, Waltham, CA, USA), used as the loading control. The antibodies used to perform the Western blot (WB) technique are described in Table 2.

### 4.7. Statistical Analysis

Data were evaluated by the Shapiro–Wilk test and are presented as mean ± standard error of the mean (SEM). Body mass and protein levels were analyzed using a one-way analysis of variance (ANOVA), followed by Tukey’s post hoc test. The dose–response curves were compared by a two-way ANOVA, with the treatment and concentration of the agonist being considered as factors. The differences among groups were determined by Tukey’s post hoc test. Statistical significance was established when *p* < 0.05.

## 5. Conclusions

We conclude that ovariectomy leads to vascular disorders associated with RAS dysregulation, determined by impairing Ang-(1-7)-mediated vasodilation in the aortic rings of ovariectomized rats. HRT with E2 and progestins was capable of attenuating this RAS imbalance. These data are of major importance considering the current paradoxes on the beneficial effects of progestin treatments on the cardiovascular system. To clarify compensatory mechanisms, further experiments will evaluate the role of eNOS modulatory sites (e.g., phospho-eNOS at Ser^1177^), as well as the role of the MrgD receptor in mediating Ang-(1-7)’s vasodilatory effects, using different pharmacological approaches (e.g., the MrgD inhibitor D-Pro7-Ang-(1-7)) [56,79,80,81].

## Figures and Tables

**Table 1:** Effect of ovariectomy and hormone therapy on body mass, uterus weight, and fat pads.

	SHAM (n)	OVX (n)	OE_2_ (n)	OE_2_ + DRSP (n)	OVX + MPA (n)
Initial Body Mass (g)	242.2 ± 5.4 (11)	247.3 ± 5.5 (15)	252.2 ± 5.3 (16)	251.0 ± 5.6 (16)	257.0 ± 6.1 (15)
Final Body Mass (g)	271.3 ± 5.8 (11)	312.0 ±7.8 *^#≈^ (15)	287.8 ± 6.0 (16)	289.3 ± 4.8 (16)	317.4 ±8.5 *^#≈^ (15)
Body Mass Gain (Δ%)	10.6 ± 1.0 (11)	26.1 ± 1.7 *^#≈^ (15)	12.2 ± 1.3 (16)	13.2 ± 1.3 (16)	23.5 ± 1.7 *^#≈^ (15)
Dry Uterus Weight (g)	12.3 ± 0.7 (11)	3.4 ± 0.1 *^#≈§^ (15)	6.0 ± 0.4 * (16)	5.8 ± 0.3 * (16)	4.8 ± 0.3 *^#^ (15)
Uterus Weight/Tibia Length (g/cm)	3.1 ± 0.3 (11)	0.9 ± 0.03 *^#≈§^ (15)	1.6 ± 0.1 * (16)	1.5 ± 0.08 * (16)	1.2 ± 0.08 *^#≈^ (15)
Parametrial Fat (g)	4.5 ± 0.4 (9)	5.0 ± 0.5 (9)	4.5 ± 0.5 (9)	5.6 ± 0.8 (9)	6.9 ± 0.8 *^$#^ (9)
Retroperitoneal Fat (g)	3.3 ± 0.5 (9)	4.2 ± 0.4 (9)	4.1 ± 0.4 (9)	4.6 ± 0.5 (9)	6.2 ± 0.5 *^$#≈^ (9)
Mesenteric Fat (g)	2.7 ± 0.1 (9)	3.2 ± 0.2 (9)	3.0 ± 0.2 (9)	3.1 ± 0.1 (9)	3.8 ± 0.3 *^#≈^ (9)
Total Fat (g)	10.6 ± 0.9 (9)	12.4 ± 1.0 (9)	11.7 ± 1.1 (9)	13.44 ± 1.5 (9)	17.0 ± 1.4 *^$#^ (9)

Values are expressed as means ± SEM and (n). * *p* < 0.05 vs. SHAM; ^$^ *p* < 0.05 vs. OVX; ^#^ *p* < 0.05 vs. OE_2_; ^≈^ *p* < 0.05 vs. OE_2_ + DRSP; ^§^ *p* < 0.05 vs. OVX + MPA. *One-way* ANOVA, followed by Tukey’s post hoc test.

**Table 2:** Primary and secondary antibodies used in the Western blot technique.

**Primary Antibodies**	**Dilution**	**Source**	**Catalog Number**	**Brand**
Anti-AT_1_R	1: 500	Mouse	SC-515884	Santa Cruz Biotechnology
Anti-AT_2_R	1: 500	Mouse	SC156014	Santa Cruz Biotechnology
Anti-eNOS	1: 2500	Mouse	BD554002	Transduction Laboratories
Anti-ACE2	1: 100	Rabbit	SC390851	Santa Cruz Biotechnology
Anti-Mas	1: 100	Mouse	SC390453	Santa Cruz Biotechnology
**Secondary Antibodies**	**Dilution**	**Source**	**Catalog Number**	**Brand**
Anti-rabbit	1: 2500	Rabbit	A0545	Sigma-Aldrich
Anti-mouse	1: 7000	Mouse	A2554	Sigma-Aldrich

## Data Availability

The data presented in this study are available upon request from the corresponding author.
